# Metrology of Short-Length Measurers—Development of a Comparator for the Calibration of Measurers Based on Image Processing and Interferometric Measurements

**DOI:** 10.3390/s24051573

**Published:** 2024-02-29

**Authors:** Pavol Kajánek, Alojz Kopáčik, Peter Kyrinovič, Ján Erdélyi, Marián Marčiš, Marek Fraštia

**Affiliations:** Department of Surveying, Faculty of Civil Engineering, Slovak University of Technology in Bratislava, 81005 Bratislava, Slovakia; alojz.kopacik@stuba.sk (A.K.); peter.kyrinovic@stuba.sk (P.K.); jan.erdelyi@stuba.sk (J.E.); marian.marcis@stuba.sk (M.M.); marek.frastia@stuba.sk (M.F.)

**Keywords:** calibration, linear measurers, systematic error determination, horizontal comparator, automatization, image processing, edge detection, non-linearity

## Abstract

For the calibration of linear scales, comparators are generally used. Comparators are devices that enable the movement of an evaluation apparatus over a calibrated scale along a linear base with high precision. The construction of a comparator includes a movable carriage that carries the device for the evaluation of the position of the given edge of the line scale relative to the beginning of the scale. In principle, it involves a camera capturing the scale of the measurer, where the position of the camera’s projection center is measured using an interferometer. This article addresses the development of a comparator assembled from low-cost components, as well as the description of systematic influences related to the movement of individual parts of the system, such as the inclination and rotation of the camera and directional and height deviations during the carriage’s movement. This article also includes an evaluation of the edge of the given scale with subpixel accuracy, addressing distortion elimination and excluding the influences of impurities or imperfections on the scale. The proposed solution was applied to linear-scale measurers, such as leveling rods with coded and conventional scales and measuring tapes. The entire process of measurement and evaluation was automated.

## 1. Introduction

In terms of the precision of levelling measurements, the quality of the level and the levelling staff itself play equally important roles. To achieve the desired precision in levelling, regular inspection of these devices must be ensured. This inspection is carried out on devices referred to as comparators. A relatively detailed classification of comparators can be found in [1].

In the past, comparators used analogue-scale readings taken through an optical microscope or through a traditional optical level; this was very time-consuming and led to a subjective interpretation of the line’s (edge’s) position on the scale [2]. Later, with the development of digital levels and CCD sensors, a transition was made to digital-scale readings, which enabled the automation of the whole calibration process. The automation of the calibration process, mainly the scale reading and the interpretation steps, led to an increased accuracy and objectivity of the results.

The development of comparators played a significant role in the advancement of both the devices themselves and other measurement techniques, especially interferometers, which serve as comparison standards. The primary categorization of comparators pertains to the calibration subject, i.e., comparators for the calibration of a levelling staff itself [1,3,4] or comparators for the calibration of an entire levelling system (level and levelling staff), in which case the calibration process is referred to as system calibration [5,6,7].

System calibration enables the calibration of an entire levelling system. The calibration principle involves comparing measurements from the level with measurements from an interferometer. Generally, system calibration requires the vertical positioning of the staff. Such positioning of the levelling staff, combined with readings taken by the level, creates conditions similar to real measurements. By comparing the correction differences between two levels with the same levelling staff, it is possible to express the differences in the systematic influences of levels [4,8,9,10,11].

For the calibration of linear measurers such as levelling staffs (classic or barcode), short-length measurers such as measuring tapes and base staffs are used as horizontal comparators [11,12]. When a linear measurer (staff, tape) is only calibrated to determine a nominal length, interferometric measurements and image processing are combined [12]. The interferometer, as an etalon, measures the length with a high accuracy, and the CCD sensor is used to determine the position of the scale division along the comparator’s baseline. There are two different concepts used in comparator construction: either (1) the CCD sensor is moved along the calibrated measurer [13] or (2) the calibrated measurer is moved along the stable CCD sensor [11]. This paper discusses horizontal comparator and their systematic errors when built according to the first concept.

Generally, it is not possible to exactly adjust (with the required accuracy) an interferometric prism to the division (tick) on the scale of the calibrated measurer. Accordingly, the relative positions of the interferometric prism and the scale division should be determined. Processing can determine this from images made by the CCD sensor (camera) mounted on the comparator’s carriage. To ensure a high accuracy for the whole process (calibration of levelling staffs is required at the micrometer level), it is necessary to eliminate the systematic influences of the following: (1) the carriage movement, (2) imperfections in the comparator geometry, and (3) the offset of the sensors used, as well as their systematic errors [14].

The calibration procedures for linear measurers (leveling staffs, tapes) or for other measuring instruments used for short distances (up to 50 m) that are used in geodesy are neither standardized nor certified. For this reason, institutions, especially universities, develop and construct their own comparators, which are generally based on the common principle of comparison, i.e., comparing the measured quantity (the distance between two lines on the scale of the calibrated measurer) with a standard (interferometer). The correctness of the calibration and the results it gives are generally ensured in two different ways: firstly, when the interferometer has traceability to the national standard (national etalon) or secondly, with the realization of a set of comparison measurements made in different laboratories (comparators) using the same set of measurers and instruments. To allow (accept) a laboratory for accreditation, it is necessary to provide acceptable results for one of the procedures described above. 

In Slovakia, the calibration of levelling staffs and system calibrations has not previously been carried out, which was one of the reasons for establishing a metrology laboratory and developing proprietary comparators. The Laboratory of Levelling Systems and Length-Measuring Instruments of the Department of Surveying at the Slovak University of Technology (STU), in Bratislava, developed and now uses both types of comparators, and it has prepared for the process of accreditation [8]. This laboratory is included in the Calibration Centre of Slovakia.

As mentioned earlier, there are several comparator solutions worldwide that differ in their reading method and in the placement of the calibrated measuring instrument. Each comparator is unique, resulting from its unique development, and has its own calibration procedure, the purpose of which is to assess the quality of the calibrated measuring instrument according to the criteria defined by the DIN18717 standard [14].

This paper presents information about the system’s design, development, and application to verify the parameters of the horizontal comparator developed to calibrate linear measurers (levelling staffs, tapes, etc.) in a horizontal position. This system verifies the linearity of the trajectory, the inclination, and the rotation of the carriage during the calibration. Using an interface, the user can determine the lateral and vertical changes in the carriage trajectory in quasi-real time and, using this information, correct the results during the calibration. The inclination sensor mounted on the carriage platform determines the carriage inclination. The rotation and the inclination of the carriage platform during the calibration result in changes in the pixel size and the orientation of the CCD images. To correct this, a projective transformation is used. 

The crucial point in the case of automated system operation and automated calibration processing is the determination of the scale division edges. The developed application identifies the division edge, eliminates the noise in the signal, enables the correction of false detections due to existing imperfections in the scale division, and finally, determines the offset (relative position) between the division edge and the interferometer prism. 

The correction of the given division of the scale is determined from the difference between the actually measured position and the reference value of the given division. The corrections can be approximated using a regression line, the parameters of which are determined using the method of least squares. The slope of the regression line corresponds to a systematic influence that significantly affects the results of the measurements. An important part of the calibration is the determination of the error of the first reading, based on which all corrections are related to the zero of the scale, which, in the case of a levelling staff, is represented by a foot. Critical values are defined for the maximum correction value, the scale coefficient, and the zero error correction in DIN 18717 [3].

As was mentioned before, each comparator is unique [7,15,16]. The advantages of the above-described development and solution lie in a fully autonomous calibration process, from the moment the levelling staff is placed on the comparator to the creation of the calibration protocol. In the design of the construction, several changes were implemented to ensure the stability of the comparator elements during measurements and to allow the calibration of other short-length measurers (measuring tapes). As part of the development, procedures were designed to verify the geometry of the comparator using the available equipment, considering the application of the results in the calibration process. The originality of the solution was examined through a patent application and subsequently granted a patent (registered at the Industrial Property Office of the Slovak Republic under No. SK289051B6).

To evaluate the edges of bars, we did not use any specialized or known edge detectors [17,18,19,20,21], or the Hough transformation [22]. Instead, we evaluated the image of the scale as a matrix in which the intensity of each pixel is registered. During data processing, the intensities of the pixels in the horizontal section (intensity profiles) over the bar were considered and evaluated. The edge evaluation process was characterized by the ability to identify damaged or contaminated edges of the component. The accuracy of the edge evaluation was verified based on the overlay. Part of the evaluation involved determining the mutual relationship between the scale plane and the CCD sensor plane, with the aim of eliminating systematic errors (tilt and rotation of the CCD sensor) through projective transformation.

The edge identification process also addressed non-ideal lighting conditions on the scale of the levelling staff, which occur due to the short object distance between the calibrated scale and the camera lens. For this reason, the algorithm automatically adjusted the intensity threshold value individually for each evaluated segment of the scale.

## 2. Materials and Methods

**Figure 1:**
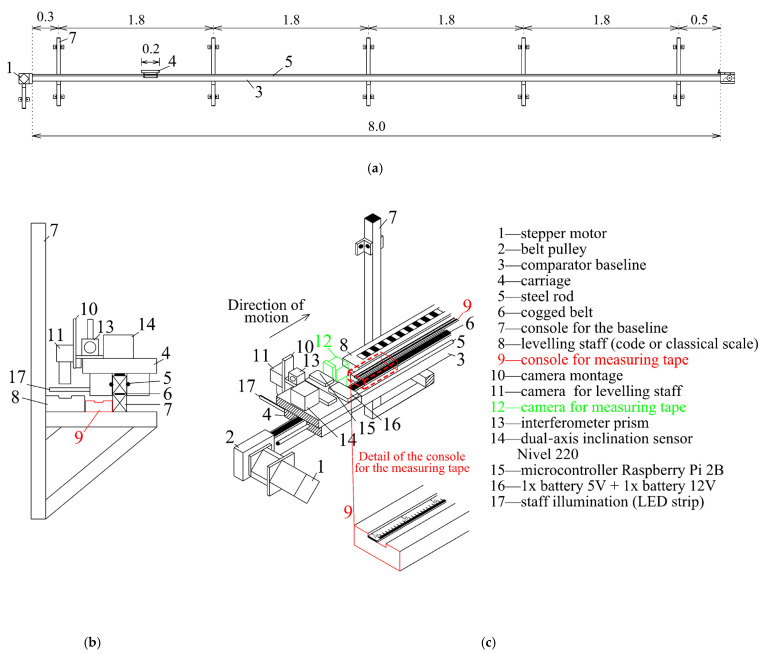
Comparator scheme—(**a**) longitudinal, (**b**) lateral, and (**c**) axonometric view.

The comparator’s structure was created using aluminum profiles (Figure 1). The base of the comparator was created using a horizontal system for carriage movement, which consisted of aluminum profiles supported by five consoles fixed in the wall. The moving part consisted of the carriage, which was set up on roller travel and whose movement was ensured by the cogged belt and the stepper motor mounted at the travel (baseline) beginning.

The motor rotation was relayed through the belt pulleys to the cogged belt, which moved the carriage. The belt adjustment was under the carriage. The carriage moved on a pair of steel rods, fixed from both sides of the aluminum profile that formed the comparator’s baseline. The carriage was set up on the rods with four roller travels, two of which had excentric bearings, which enabled the rectification of the carriage setup. The baseline length of 8 m limited the travelling length of the comparator. At the end of the baseline, inductive sensors were installed on both sides to stop the carriage movement.

**Figure 2:**
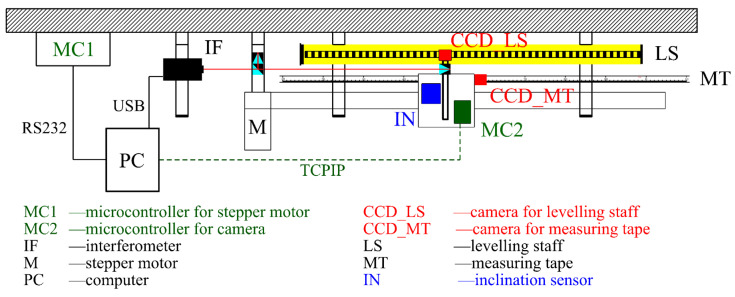
Comparator management scheme.

For the calibration of linear measurers, two comparator solutions were employed: (1) a comparator with the evaluation device (camera) moving above the linear measurer and (2) a comparator with a moving calibrated measurer alongside the evaluation device, which remained fixed. Due to the limitations of this article, we focused on the first type of comparator, where the calibrated measurer is in a horizontal position and the camera is travelling above the calibrated measurer (Figure 1). Figure 1 and Figure 2 provide a schematic representation of a horizontal comparator. Due to the different scale levels of levelling staffs and tapes, separate cameras (Figure 1–item 12) were used for levelling staffs and measuring strips. The cameras were positioned above the invar tape of the levelling staff or the measuring strip. Throughout the process, it was not necessary to refocus the camera, which was important for achieving the required accuracy for the image processing. The measuring tape was placed on a separate base so that separate brackets, different from those used for the levelling staff, were employed. For clarity, these brackets are not included in the figures. Due to the limited length of the comparator, the calibration of tapes was performed on 6 m sections with a 1 m overlap.

The stepper motor managed the carriage movement and was autonomously stopped with the camera over the following scale division edge (Figure 2). The camera montage ensured a normal position of the projection axis relative to the plane of the calibrated scale (for example, the invar tape). Managing the camera was possible by using the microcontroller Raspberry Pi, which managed the capturing of images and their transfer to the central PC. The microcontroller power was provided by a battery installed on the carriage. To create excellent conditions for image capture, extra lighting was installed from both sides of the camera using two LEDs positioned at the height of the camera objective. The camera inclination was measured by two Leica Nivel 220 axial inclination sensors, with the Y-axis oriented parallel to the comparator baseline and the X-axis perpendicular to them, defining the right-oriented coordinate system. The interferometer prism was mounted at the same console to achieve a stable (fixed) offset between the central point of the CCD sensor and the central point of the prism.

The calibration of linear measurers with scales is based on the comparison of the actual position of the scale divisions (ticks) with their reference position. The combination of interferometric measurements and image processing determines the actual position of the division. The resulting image processing is determined by the position of the division (their edges) relative to the central point of the camera image. The movement of the camera and its central point are measured using the interferometer. The whole process is managed fully autonomously; the central PC produces the commands both for the microcontroller managing the stepper motor (through the serial port) and for the microcontroller managing the camera (using the TCPIP protocol).

### 2.1. Identification and Elimination of Systematic Errors

To achieve the required accuracy for the calibration results at the micrometer level, it is necessary to know and eliminate the systematic errors of the comparator. Comparator errors related to the imperfection of the movement are transferred to the evaluated position of the scale divisions; therefore, their elimination is necessary. The camera’s movement above the scale generates errors caused by non-linear movement and changes in the camera’s orientation in space. Furthermore, it is necessary to consider corrections from the camera’s tilt, its rotation around the shot axis, and changes in the subject’s distance from the camera in the calculation.

#### 2.1.1. Verification of the Comparator’s Baseline Linearity

**Figure 3:**
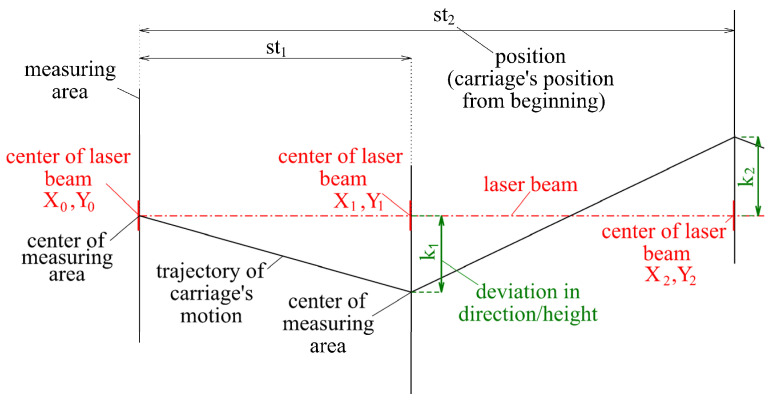
Principle of evaluation of the deviations in the laser beam position in the lateral and vertical direction.

The horizontal comparator of the calibration laboratory of the Department of Surveying uses the construction principle, where the camera is moved along the calibrated measurer. The actual position and orientation of the camera during its movement along the calibrated measurer are given by the actual position and orientation of the carriage, which depend on the geometry of the baseline. Accordingly, it is important to know and verify the complex geometry of the carriage trajectory, which should be linear and parallel to the calibrated measurer, and during carriage movement, no changes should occur in its inclination or orientation. Due to imperfections in the geometry of the baseline, the trajectory of the carriage was not ideal, and corrections from the camera offset and orientation needed to be applied during the calibration (Figure 3). The inclination of the carriage was measured using the implemented inclination sensor Leica Nivel 220. The imperfections in the baseline geometry were determined using the newly developed system, which used a laser beam as an etalon (Figure 3).

**Figure 4:**
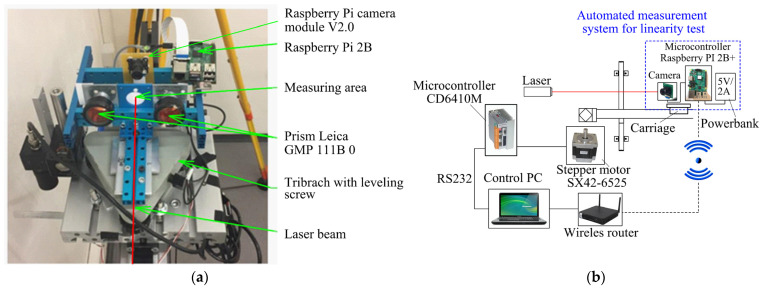
Measuring system for verification of the comparator’s baseline linearity: (**a**) main components of the measuring system; (**b**) operational scheme of the system.

**Figure 5:**
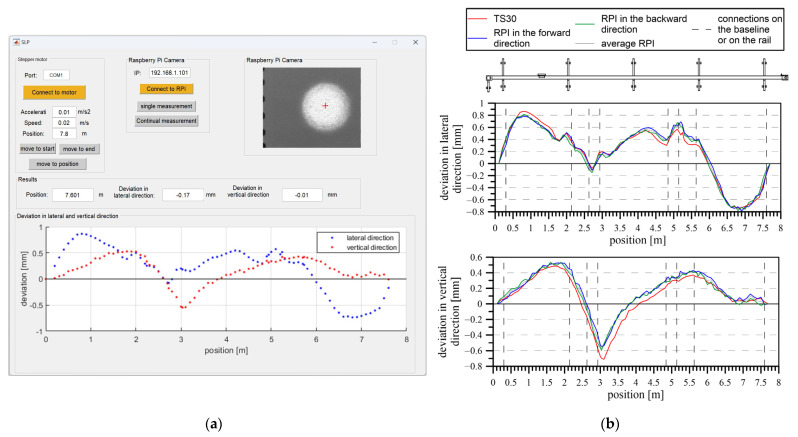
Measuring system for the verification of the comparator’s baseline linearity: (**a**) the graphical interface of the system; (**b**) the deviation from the baseline in the lateral and vertical directions.

The entire system was mounted on a trolley (see Figure 4a). The position of the laser beam on the measured surface was determined through image processing using a Raspberry Pi Module v2 camera equipped with a Sony IMX219 image sensor boasting a resolution of 3280 × 2464 pixels. The camera was positioned behind the measuring surface on a shared structure, ensuring a stable and consistent geometry for both the camera and the measuring surface. A Raspberry Pi microcontroller managed the control of the camera. The captured images were then transmitted to a central computer for data processing, employing the TCPIP protocol for communication between the central PC and the microcontroller (refer to Figure 4b). This system featured a graphical interface (see Figure 5a) that enabled users to assess these deviations in quasi-real time (6 s per point). This system’s objective was to conduct a swift and regular assessment of the linearity of the comparator movement. 

The ideal trajectory of the base was represented using a laser beam. Directional and height deviations from the ideal trajectory were displayed as a change in the position of the beam track, which was evaluated by image processing. The system evaluated the direction and height deviations in the selected stations. Deviations in the comparator’s baseline determined by the system were from the interval of −0.8 mm to +0.9 mm in the lateral direction and between −0.7 mm and +0.6 mm in the vertical direction (Figure 5b). The results were verified through terrestrial measurements using the polar method, implemented with the universal measuring station Leica TS30. The accuracy and utility of the system were demonstrated through consistent results from repeated measurements and a comparison with the Leica TS30 universal measuring station (Figure 5b).

#### 2.1.2. Correction of the Interferometric Measurement from the Camera Inclination and Rotation

The final position of the detected edge $l_{b}$ (Equation (1)) was the interferometrically determined position of the projection center of the camera $l_{\mathrm{if}}$ and the distance $l_{\mathrm{im}}$ of the edge of the scale division from the main point of the image, which was evaluated by image processing. The final position of the detected edge is given by the following equation:

(1) $$l_{b}=l_{\mathrm{if}}+l_{\mathrm{im}},$$

**Figure 6:**
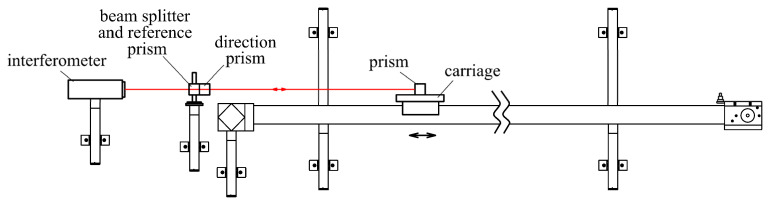
Scheme of the interferometric measurement.

Interferometric measurements play a crucial role in determining the precise position of the camera’s center (Figure 6). Since the camera does not stop directly above the edge of the scale bar, it is necessary to evaluate the distance between the main point of the image and the edge of the bar by processing the image. When evaluating this distance, changes in the inclination and rotation of the carriage, which affect both the camera and the desired calibration accuracy, must be considered. This correction quantifies the actual distance between the camera projection center and the detected edge, measured along the baseline of the comparator.

**Figure 7:**
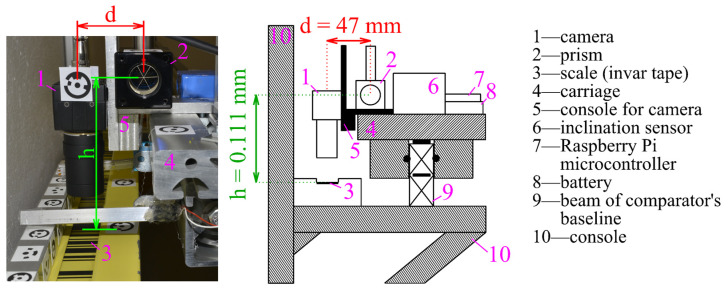
Photo and side view of the system (d—the distance between the prism and the camera center; h—the difference in the height of the camera CCD sensor and the scale).

Due to the offset between the camera and the prism, the interferometric measurements should be corrected by the inclination and rotation of the carriage, determined during its movement (Figure 7). It is mainly the inclination of the camera in the longitudinal direction (along the baseline of the comparator) that significantly affects the interferometric measurements.

**Figure 8:**
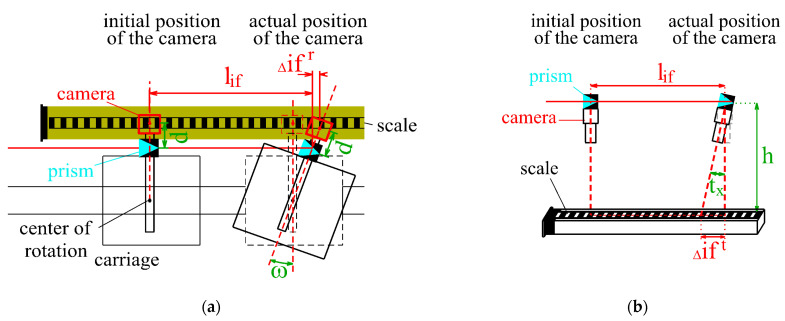
Determination of the correction due to camera movement: (**a**) camera rotation (nadir view) and (**b**) camera inclination (side view).

As a result, the interferometric measurement $l_{\mathrm{if}}$ should be corrected by the value $\Delta if^{t}$ (Figure 8b), which can be determined using the measured camera inclination $t_{x}$ and the difference h between the height of the interferometric prism and the height of the calibrated scale (invar tape):

(2) $$\Delta if^{t}=h\cdot tg\left(t_{x}\right),$$

The carriage rotation during the calibration also affects the interferometric measurements significantly. The correction of the interferometric measurement $\Delta if^{r}$ should be determined (Figure 8a) using the camera rotation ω and the distance d measured between the interferometric prism and the central point of the camera’s CCD sensor.

(3) $$\Delta if^{r}=d\cdot tg(\omega ),$$

Due to the common montage (fixation) of the camera and the interferometric prism on the carriage, it can be assumed that the carriage and the camera had the same rotation during the calibration. The camera rotation was calculated during image processing using the angle between the regression line of the detected edge and the longitudinal axis of the calibrated scale (invar tape). To determine the angle, the edge with the smallest distance to the image’s central point was used.

**Figure 9:**
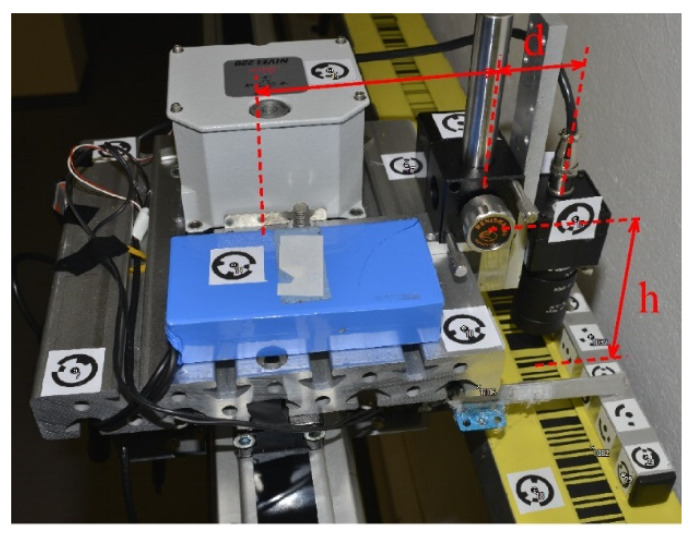
Relationship between the CCD sensor, the interferometric prism, and the scale.

The distance d between the CCD sensor’s center and the prism, as well as the height difference h between the height of the CCD sensor and the height of the scale, was determined with a photogrammetric method using two convergent images, which required the selection and marking of identical points on the system’s construction (Figure 9). The position of the CCD sensor inside the camera housing was given by the producer’s data sheet. All the determined corrections expressed the effect of the relative changes in carriage inclination and rotation during the calibration according to their initial position, which was the position over the first detected edge on the scale.

### 2.2. Determination of the Position of Scale Divisions

**Figure 10:**
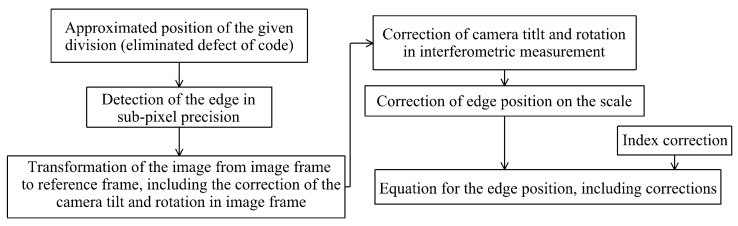
Determination of the scale division corrections.

The principle of determining the position of divisions was developed first for the code scales of levelling staffs. This principle was subsequently tested for other measurers, such as levelling staffs with classical divisions and measuring tapes. First, the whole process of determining the position of the scale divisions was described, and from it, the corrections were determined (Figure 10). Finally, the results of the usage of the procedure for other scales are presented.

**Figure 11:**
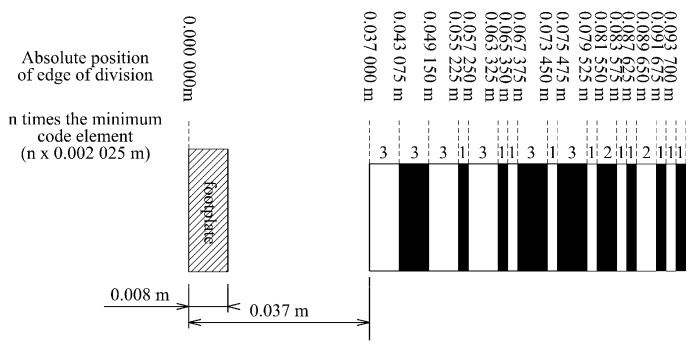
The absolute position of the division edges of the Leica levelling staff.

**Figure 12:**
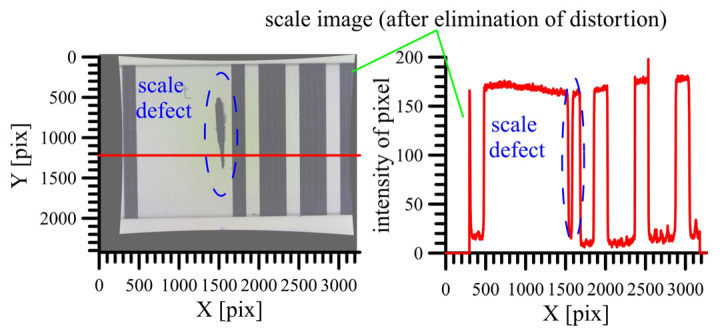
Detection of edges and determination of their approximate position on the scale.

The position of the scale divisions was determined using image processing and interferometer measurements. First, the approximate position of the division was determined, and the camera’s field of view was set over the detected division. In this position, the position of the cameras (their projection center) was precisely defined using interferometric measurements. When capturing an image, the approximate position of the scale divisions (their edges) was known. Comparing this position with the manufacturer’s reference value (Figure 11) could eliminate possible false detections, for example, scale defects (Figure 12).

Furthermore, the dividing edges were determined with subpixel precision. Due to changes in the tilt and rotation of the camera position during calibration (carriage movement), the size of the pixels changed according to their distance from the center of the image. This was corrected using a projective transformation, which transformed all the images taken during calibration to the plane of the calibrated scale. Due to changes in the tilt and rotation of the camera position during calibration, the interferometric measurement results also needed to be corrected. To determine the corrections, the slope sensor signal and the orientation of the regression line were approximated using the dividing edges.

In the case of scales that do not have a defined zero point (levelling staff), all measurements and corrections were fixed to the first evaluated part of the scale. Their position, according to the scale’s zero point (zero value), should be determined as an index correction.

Calibration uses information about the reference values of the scale divisions. These are given (1) in the form of the absolute distance between the division and the zero point of the scale (subtraction) or (2) in the form of a code that contains the basic interval between the divisions and their color. Using this information, the absolute reference position of each division on the scale was calculated. Each manufacturer has its own code; however, these do not change anymore, and are instead defined for a larger scale range. For shorter scales, a smaller part of the code is used.

The most calibrated measurers are levelling staffs, the scale of which is built on the basis of an 8-bit code [23]. There are four manufacturers of levelling sticks on the market (Leica, Trimble, Topcon, and Sokkia), and they all use different codes. For example, the Leica levelling staff code consists of sections whose width is an integral multiple of the smallest color section of 2.025 mm (Figure 11) [24]. Leica uses the same code for all the levelling staffs it produces.

### 2.3. Detection of the Edge of the Scale Division

The positions of the scale divisions were determined using image processing. The approximate position of the divisions and their edges were defined using interferometric measurements and their comparison with reference scale values. To minimize the influence of camera lens distortion, it was important to set the camera very close to the edge of the scale that was being evaluated. Therefore, the camera moved autonomously during the entire calibration. When the camera movement was stopped, its approximate position above the scale was determined. According to the camera’s field of view, the number of visible scale edges was determined, and the exact position of the evaluated edge was determined using image processing.

The edges of the scale divisions were identified based on a significant change in the intensity, measured along the longitudinal section of the scale (Figure 12). The algorithm can detect various scale defects as edges. These were eliminated through a comparison of all the detected edges, with the reference position of the edge on the scale given by the producer. This comparison served only to exclude false detected edges on the scale.

Due to the imperfect illumination of the camera’s field of view and the close position of the camera to the scale, the intensity of the pixels was not the same, and it increased according to the distance of the pixel from the center of the image. The central part of the image was less illuminated, despite the use of additional illumination in the field of view of the camera. Accordingly, the image-processing algorithm used a different threshold to detect edges in a different part of the image. The exact position of an edge was determined with subpixel accuracy. For each edge, a slice of the image was created with a range of pixels backward and 50 pixels forward from the detected edge, and the actual threshold was calculated for both the black and white (yellow) regions in that field. A new threshold value $t_{e}$ was calculated for each section (Equation (4)), which was adapted to the current illumination of the scale.

(4) $$t_{e}=\frac{\frac{\sum_{j=1}^{30}I_{j}}{30}+\frac{\sum_{j=71}^{100}I_{j}}{30}}{2},$$

The position of the edge was determined using a regression line calculated from 1000 points resulting from edge detection in 1000 parallel slices made through the image. The regression line parameters were determined using the method of least squares. The direction of the regression line was used to calculate corrections for camera rotation changes.

### 2.4. Projective Transformation of Images into the Reference Plane

**Figure 13:**
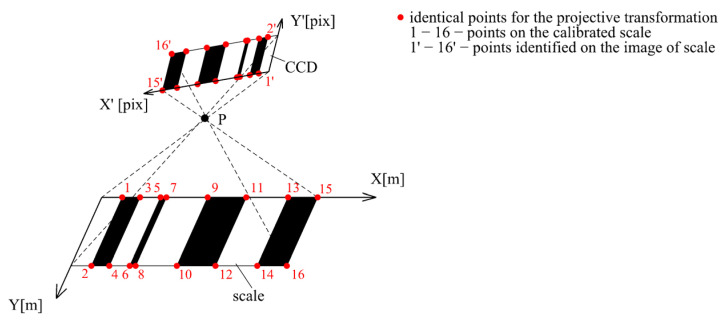
Projective transformation of the image to the reference plane.

The inclination of the camera caused a change in the pixel size of the image. To eliminate this, a projective transformation was applied, which defined the relationship between the camera’s CCD sensor and the plane of the scale (invar tape). The projective transformation did not serve to calculate the exact edge position; rather, it was only used for the removal of the errors due to the camera’s tilt. The parameters of the projective transformation (three translations and three rotations) were calculated for each image using identical points (Figure 13). The identical points were defined by the intersection of the edges and the invar tape edge (defined by the visible borderline of the invar tape). The reference coordinates of these points were calculated using the reference values of the scale code and the width of the visible part of the invar tape, which was 22 mm. The image coordinates of these points were defined by the intersection of the regression lines representing the division edges and the invar tape borderline, which was visible on the image.

**Figure 14:**
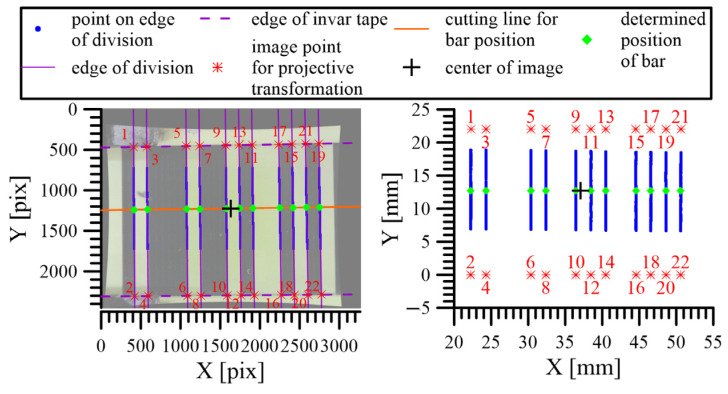
Determination of the edge position in the plane of the invar tape.

Using the projective transformation, all the edges were transformed to the plane of the invar tape (Figure 14, left). In each transformed image, the distances between the image’s center point (Figure 14, black cross) and the edges (Figure 14, green dots) were measured in the direction of the longitudinal section (Figure 14, purple line). The position of the image’s center point in the plane of the invar tape was determined using interferometric measurements.

### 2.5. Evaluation of Corrections of Scale Divisions of a Calibrated Measurer

**Figure 15:**
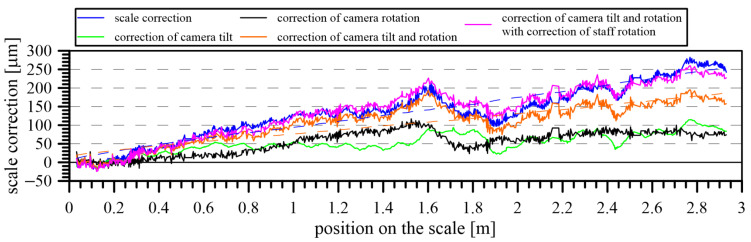
Corrections of the scale calibration before and after the application of corrections due to the camera inclination and rotation.

The calibration results included a significant influence of camera position changes (Figure 15) caused by the camera’s imperfect movement along the calibrated scale. After removing this influence, the results were free from systematic errors. First, the actual tilt and roll of the camera were determined using the coordinates of the camera’s center point, the interferometric position, and the height difference of the CCD sensor and the scale.

**Figure 16:**
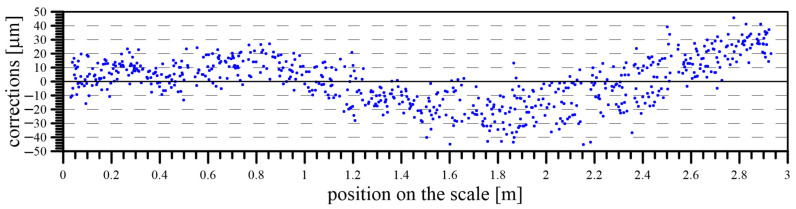
Final scale corrections of the calibrated levelling staff.

When all the systematic errors (caused by the inclination and rotation of the carriage and an imperfect position of the staff) from the result of the calibration were eliminated, the final results of the scale calibration in the form of corrections for the given scale positions were determined (Figure 16).

**Figure 17:**
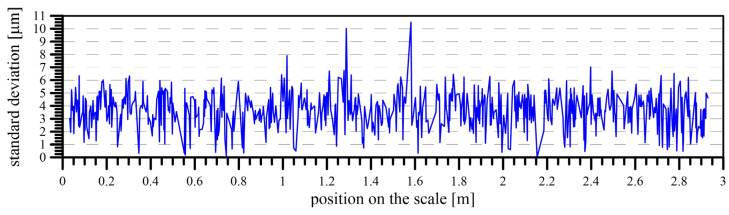
The standard deviation of scale corrections.

After the elimination of all the systematic influences that affected the calibration process, the quality of the calibration was expressed and evaluated. This was expressed using the standard deviation of the correction, determined by the calibration for each division on the scale. The possibility of calculating the standard deviation of each division is given, with multiple determinations of this correction for each division. Due to this situation, when more divisions of the calibrated scale were present in each image, their actual position was determined more times. Generally, each division was visible in 6–7 images, which enabled the calculation of the final result of the calibration as the average value of this series, including their standard deviation (Figure 17).

**Figure 18:**
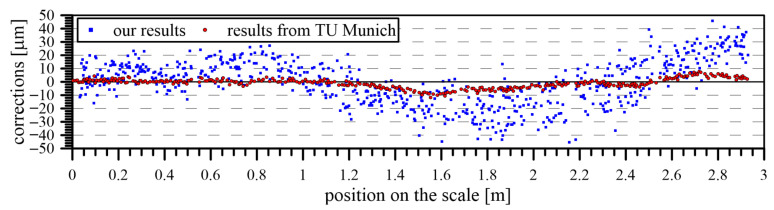
Comparison of calculated corrections with corrections from the calibration laboratory at TU Munich.

A comparison of the calculated corrections between our comparator and the comparator of TU Munich [25] can be observed in Figure 18. The reasons for the selection of this comparator were the availability of the protocol for the used levelling staff and the known high quality of the comparator itself, which can be classified among the most accurate in the world. By comparing the calculated corrections of the components with the corrections obtained from the calibration protocol of the staff created at TU Munich, the same scale factor of the staff was determined. The correction profiles were similar in both protocols, but the amplitude of corrections in our results was twice as large. This could be attributed to the different types of equipment used to evaluate the scale components. TU Munich uses an electro-optical microscope (Zeiss MPV Compact) in combination with commercial software for edge identification during evaluation [25]. It should also be taken into account that there was a 7-year time interval between the performed measurements, during which the levelling staff was used for standard measurements in terrain.

As was mentioned before, the laboratory, including the comparators, was actually prepared for the accreditation process, for which it is necessary to have the results of comparative measurements. Therefore, the conduction of comparative measurements with a broader range of calibration laboratories is planned, which will help us to better evaluate the results and their quality in the future. As part of this comparison, a short time interval will be ensured between calibrations, and we will also exclude the influence of the levelling staff usage on the calibration results.

Our goal is to achieve accreditation of the laboratory. The first step on this path is to obtain a patent of the calibration procedure, which has already been registered at the Industrial Property Office of the Slovak Republic under No. SK289051B6. We have received support from the national calibration center in geodesy for the accreditation of our laboratory, including the calibration of short-length measurers. At this stage, we do not intend for this procedure to become a part of the national or international standard.

### 2.6. Determination of the Scale Index Correction

All the corrections were determined relative to the first division on the scale. In many cases, the initial division (zero reading) of the scale was not visible or was represented by a special element (e.g., the base of the levelling staff). In this situation, the calibration started at the first visible (accessible) division and the corrections of all the divisions were determined with respect to the position of the first visible division of the scale. The absolute position of the first division should be determined to obtain the correct absolute position of all scale divisions. This procedure is known as determining the scale index correction $v_{G}$. In fact, it is about determining the actual value of the distance between the foot of the levelling staff and the first visible edge (division) on the scale and comparing them with the reference value (Figure 18). The index correction of the calibrated scale was determined as follows:

(5) $$v_{G}=l^{r}-l^{m}.$$

The reference value of the distance $l^{r}$ is given by the reference position of the edge (division), which is derived from the code of the levelling staff.

To determine the actual position of the first visible edge $l^{m}$, a plane-parallel glass plate was placed exactly on the footplate, on which a grid of crosses with a very precisely determined position was etched. The camera was moved over the glass plate, where the image was captured, and the interferometer and tilt sensor data were registered. Then, the carriage with the camera was moved to the first edge of the scale, and its position was determined.

**Figure 19:**
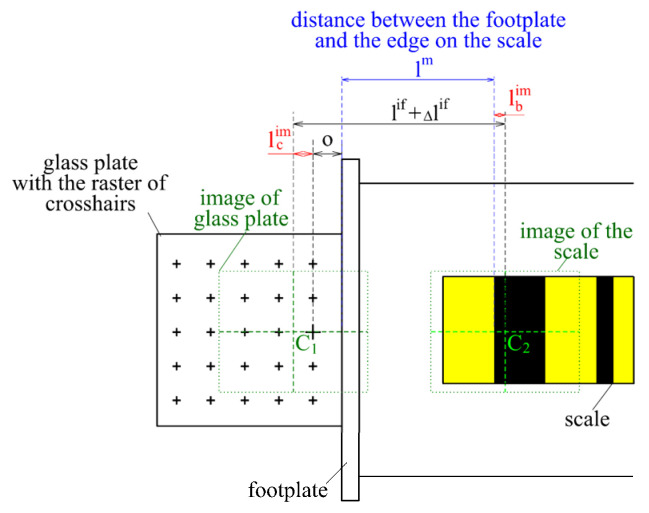
Determination of the distance between the edge and the footplate.

The change in the camera position $l^{\mathrm{if}}$ was defined using an interferometer (Figure 19). The distances between the center point of the C1 image and the edge $l_{b}^{\mathrm{im}}$ and between the center point of the C2 image and the crosshair $l_{c}^{\mathrm{im}}$ were determined using image processing. Finally, the offset o between the crosshair and the footplate of the levelling staff were subtracted.

Corrections for the interferometer measurements $\Delta l^{\mathrm{if}}$ were calculated based on the measured camera tilt. The actual value of the distance $l^{m}$ between the edge on the scale and the footplate was determined using the following equation:

(6) $$l^{m}=(l^{\mathrm{if}}+\Delta l^{\mathrm{if}})-o+(l_{b}^{\mathrm{im}}-l_{c}^{\mathrm{im}}),$$

**Figure 20:**
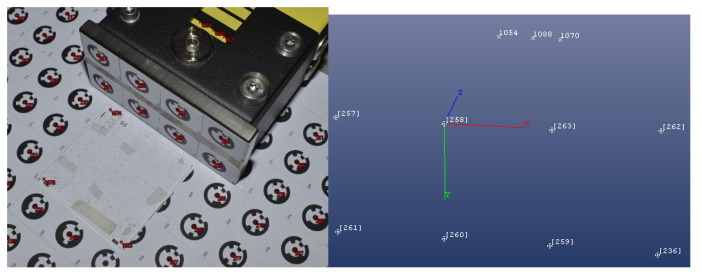
Determination of the scale index correction using multi-image photogrammetry.

An independent measurement was performed to validate the procedure developed for the determination of the scale index correction. It was decided to use multi-shot close-range photogrammetry [21], during which a series of convergent images of the footplate and scale were taken. For this reason, the footboards and their surroundings were marked with a set of control points using coded characters (Figure 20). Furthermore, the 3D coordinates of the marks on the footplate and the edge points (1054, 1088, and 1070) were determined. Using the points on the footplate (258, 260, and 263), the actual position of the footplate plane and the distance between the edge of the first bar and this plane in the perpendicular direction to the plane were determined.

For data processing, the software PhotoModeler UAS Build 2017.1.1.2199 was used. The scale of the model was defined using the same glass plate with crosshairs, which was also used in the first measurement. The local coordinate system used for the calculation was set into the footplate (XY plane), with the Z-axis perpendicular to this. Equation (7) provides the distance between the footplate and the edge of the division on the scale, measured in the normal direction:

(7) $$v_{G}=Z_{E}-Z_{F}-t_{p},$$

where

$Z_{E}$ is the coordinate of the edge,
$Z_{F}$ is the coordinate of the footplate, and
$t_{p}$ is the thickness of the used coded signs.

Based on the comparison (Table 1), it could be concluded that the difference between the two methods was small and varied between −13.6 µm and 4.0 µm. The higher uncertainty in the photogrammetric measurements could have been caused by the manual measurement of the image coordinates of the points representing the edge position. The verification results showed that the procedure developed for scale index determination was correct and worked with the required accuracy.

## 3. Discussion

To ensure the required high accuracy of a calibration, it is necessary to analyze the comparator’s systematic errors, which are caused by the imperfection of the comparator structure and its elements. In the calibration of linear measurers, an interferometer is used as the etalon for the determination of the camera position with a high accuracy. A camera mounted on the carriage provides images of the calibrated scale. The distance between the calibrated edge (division) and the interferometric prism can be determined using these images. To obtain calibration corrections with the required accuracy, the main task is to ensure the responsible determination of the relative geometry parameters of the comparator baseline, the interferometer’s laser beam, the calibrated measurer’s longitudinal axis, and the camera axis. Due to the non-parallel position of the calibrated scale, the comparator’s baseline and the non-perpendicular position of the camera to the scale plane were considered in the systematic errors of the calibration corrections, the values of which changed during the movement of the camera (carriage). The non-linearity of the steel rod used for the carriage movement resulted in lateral and vertical changes in the carriage trajectory as well as its inclination in both directions and its rotation. Because of the fixed montage of the camera on the carriage, the position and orientation of the camera changed in the same way during the calibration. 

To verify the linear position of the rods, a system was developed that enabled the autonomous determination of the lateral and vertical changes of the carriage’s trajectory in quasi-real time (with a 6 s time shift). The system used a laser beam and determined the changes in position in both directions. The results produced by the system were verified through terrestrial measurements, and differences of ±0.1 mm were accepted in the lateral direction and ±0.3 mm in the vertical direction.

The camera inclination and rotation during the carriage movement resulted in different pixel volumes inside the images. An algorithm that corrected this using a projective transformation was developed and tested. The application of the developed algorithm allowed the images taken by the camera in the actual position to be transformed into the reference plane of the calibration procedure, which was the plane of the calibrated scale. The parameters of the projective transformation were determined using identical points located in the corners of the divisions visible on the image.

The camera inclination and rotation also systematically influenced the distances measured by the interferometer. Due to the given offset of the camera’s central point and the interferometric prism, corrections of these distances were calculated according to the actual camera position. The direction of the detected division edges (represented by a regression line) was used to calculate these corrections, as well as the data of the dual-axis inclination sensor mounted on the carriage.

The calibration resulted in a calibration certificate, in which the client can obtain all the necessary information about the calibration and how to use the calibration results. For the limited range of this article, the calibration certificates are presented as follows: a calibration certificate for a levelling rod with a classic scale is presented in File S1, a calibration certificate for a levelling staff with a coded scale is presented in File S2, and a calibration certificate for a measuring tape is presented in File S3.

The proposed solution for evaluating the position of a workpiece edge using image processing and interferometric measurements has been submitted and approved as a patent. This solution is actively used in the calibration of coded levelling staffs and is currently undergoing testing on levelling rods with traditional divisions and measuring bands. In future work, we plan to conduct comparative measurements with other laboratories and prepare the documentation for laboratory accreditation. In addition to the mentioned horizontal comparator, we have also developed a vertical comparator that calibrates the entire levelling system, meaning that both the levelling staff and the level are calibrated together. The resulting corrections apply to the entire levelling system. By comparing the results between comparators, we can eliminate the systematic influence of the levelling staff itself from the corrections for the entire levelling system, thereby evaluating the systematic errors of the level itself. The regular calibration of instruments and gauges is important for improving the quality of the instruments themselves and enhancing the quality of the results in geodesy and other activities where these instruments are used.

## 4. Conclusions

This paper dealt with calibrating linear measurers using image processing and interferometric measurements. Parallel to the development of a comparator, an application was developed to automatically manage the whole calibration process. This application enables the autonomous movement of the carriage and the camera to the scale divisions, the collection of images, and data collection from all the sensors used to monitor the physical conditions in the comparator’s surroundings and the stability of the comparator’s construction. The quality of the calibration using the comparator and the managing application was verified in a series of test calibrations of a levelling staff, but this comparator and application could be used to calibrate any linear measurers.

This paper provides complex analyses of the systematic influences and error sources caused by the imperfection of comparator geometry. It also describes the designed algorithms and system, which were developed for the determination of the actual changes in the camera position during its movement along the calibrated scale. The designed algorithms include procedures for edge detection, detection of the laser beam center, and identification of the scale divisions, including the identification of falsely detected divisions, which occur due to scale damage. The described model of data processing was tested and verified through the calibration of a series of levelling staffs with barcodes.

## 5. Patents

The development of the horizontal comparator represents an innovative solution (invention) that became the subject of a patent registered at the Industrial Property Office of the Slovak Republic under No. SK289051B6, kept in the register of inventions under the designation G01B 11/02 (ÚPV SR, 2023). The subject of the invention is a device and a procedure for evaluating the edge of the scale division of a linear scale that evaluates the position of the edge of the scale division of a calibrated measurer concerning the standard with the required accuracy at the micrometer level.

## Figures and Tables

**Table 1:** Comparison of scale index corrections determined using calibration and photogrammetry.

Index Correction [µm]			Differences between Methods
	Measurement on Comparator	Convergent Imaging of Footplate and Invar Tape	
1st measurement	24.5	29.1	−4.6
2nd measurement	28.1	24.1	4.0
3rd measurement	39.2	27.9	11.3
4th measurement	31.9	38.9	−7.0
5th measurement	31.7	45.3	−13.6
Average	31.1	33.1	-
Standard deviation	4.9	8.8	-

## Data Availability

Data are contained within the article.

## Supplementary Materials

The following supporting information can be downloaded at: https://www.mdpi.com/article/10.3390/s24051573/s1.

## References

[1] Baričević S., Staroveški T., Barković Ð., Zrinjski M. (2023). Measuring Uncertainty Analysis of the New Leveling Staff Calibration System. Sensors.

[2] Vyskočil Z., Lukeš Z. (2015). Horizontal comparator for the system calibration of digital levels–realization at the Faculty of civil engineering, CTU Prague and in the laboratory of the Department of survey and mapping Malaysia (JUPEM) in Kuala Lumpur. Geoinform. FCE CTU.

[3] Wu C.T., Chen C.S., Chang M.W. (2013). Uncertainties in the Calibration System for Invar Leveling Rods. Tamkang J. Sci. Eng..

[4] Wasmeier P., Foppe K. A new CCD-based technique for the calibration of levelling rods. Proceedings of the XXIII International FIG Congress.

[5] Takalo M., Rouhiainen P. (2004). Development of a system calibration comparator for digital levels in Finland. Nord. J. Surv. Real Estate Res..

[6] Woschitz H., Brunner F.K. (2003). Development of a vertical comparator for system calibration of digital levels. Austrian magazine for surveying and geoinformation. ZfV Mag. Geod. Geoinf. Land Manag..

[7] Woschitz H., Brunner F.K., Kopáčik A., Kyrinovič P. System calibration of digital levels–experimental results of systematic effects. Proceedings of the INGEO2002, 2nd Conference of Engineering Surveying.

[8] Kajánek P., Kopáčik A., Ježko J., Erdélyi J. (2020). The impact of systematic errors of levels on the results of the system calibration. Advances and Trends in Geodesy, Cartography and Geoinformatics II.

[9] Szczutko T. (2011). Invar rod calibration on vertical comparator executed in the geodesy metrology laboratory of the AGH University of science and technology in Krakow–Poland with use of computer-aided image analysis. Rep. Geod..

[10] Woschitz H., Gassner G., Ruland R. (2007). SLAC vertical comparator for the calibration of digital levels. J. Surv. Eng..

[11] Woschitz H., Brunner F.K., Heister H. Scale Determination of Digital Levelling Systems using a Vertical Comparator. Proceedings of the FIG XXII International Congress.

[12] Zhao M., Huang Q.H., Zhu L.J., Qiu Z.M. (2015). Automatic laser interferometer and vision measurement system for stripe rod calibration. Metrol. Meas. Syst..

[13] Gassner G.L., Ruland R.E. Investigation of Leveling Equipment for High Precision Measurements. Stanford Linear Accelerator Center (SLAC). Proceedings of the American Congress on Surveying and Mapping.

[14] (1996). Präzisions-Nivellierlatten.

[15] Urban R., Štroner M., Braun J. (2015). Special electronic distance meter calibration for precise engineering surveying industrial applications In Optical Measurement Systems for Industrial Inspection IX.

[16] Braun J., Štroner M., Urban R., Dvořáček F. (2015). Suppression of systematic errors of electronic distance meters for measurement of short distances. Sensors.

[17] Canny J. (1986). A Computational Approach to Edge Detection. IEEE Trans. Pattern Anal. Mach. Intell..

[18] Lim J.S. (1990). Two-Dimensional Signal and Image Processing.

[19] Parker J.R. (1997). Algorithms for Image Processing and Computer Vision.

[20] Ziou D., Tabbone S. (1998). Edge detection techniques-an overview. Pattern Recognit. Image Anal. C/C Pattern Recognit. Image Anal..

[21] Luhmann T., Robson S., Kyle S., Boehm J. (2007). Close Range Photogrammetry.

[22] Yazdani A., Aalizadeh H., Karimi F., Solouki S., Soltanian-Zadeh H. Sub-pixel X-marker detection by Hough transform. Proceedings of the IEEE 2018 25th National and 3rd International Iranian Conference on Biomedical Engineering (ICBME).

[23] Ingensand H. (1999). The evolution of digital levelling techniques-limitations and new solutions 1999. The Importance of Heights.

[24] Fischer T., Fischer W. (1999). Manufacturing of High Precision Leveling Rods 1999. The Importance of Heights.

[25] Heister H. (1988). For the automatic calibration of geodetic length measuring instruments. Surveying Course.

