# Supplementary material for: Metrology of Short-Length Measurers—Development of a Comparator for the Calibration of Measurers Based on Image Processing and Interferometric Measurements
Source: Sensors (Basel). 2024 Feb 29;24(5):1573. doi: 10.3390/s24051573 (PMC10934804; doi:10.3390/s24051573)
Supplement: Supplementary file 1 [file sensors-24-01573-s001.zip › File S1-clasic_scale.pdf]

Calibration certificate no. 09/2022

Leveling staff (type, no.):
GPLE3N,, no. 47068
Calibration range [m]:
0,12043 - 2,87934
Comparator:
Horizontal comparator
Contract:
[redacted]
Date:
3.9.2022

Laboratory of metrology of short lengths
Slovak University of Technology in Bratislava
Faculty of Civil Engineering
Department of Surveying
Radlinského 11
810 05 Bratislava
tel. [redacted]
email: alojz.kopacik@stuba.sk

Coefficient of expansion (standardized value):
$\alpha_{T_N} = 0{,}36\, ppm \cdot °C^{-1}$

Position of the leveling staff during calibration:
horizontal

Determinaion of scale factor:

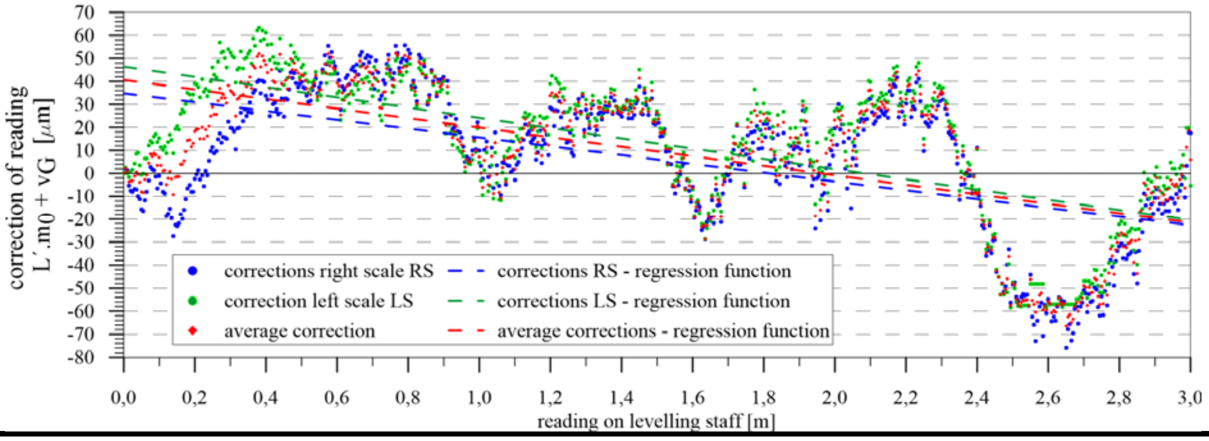

Scale factor: $\boldsymbol{m_0 = -20.73 \pm 0{,}02\ ppm}$ $\boldsymbol{T_0 = 20{,}8°C}$ $\boldsymbol{v_G = 12 \cdot 10^{-6}\ m}$

Reading on the levelling staff after correction:

$$L = L' \cdot [1 + m_0 + \alpha_T \cdot (T - T_0) \cdot 10^{-6}] + v_G$$

$L´[m]$	measured value	m_0	scale factor
$T[°C]$	temperature during measurement	$\alpha_T[\mathrm{ppm} \cdot °C^{-1}]$	coeficient of expansion
$T_0[°C]$	temperature during calibration	$v_G[m]$	index correction

Determination of scale factor:

1. right scale:

scale factor:		Average temperature	20,8 °C
-19.16 ppm	± 0,02 ppm	Average atmospheric pressure:	999,0 hPa
		Average deviation of measurements:	± 1.92 μm

2. right scale:

scale factor:		Average temperature	20,9 °C
-22.30 ppm	± 0,03 ppm	Average atmospheric pressure:	999,0 hPa
		Average deviation of measurements:	± 2.05 μm

Average from both scales:

scale factor:		Average temperature	20,8 °C
-20.73 ppm.	± 0,02 ppm	Average atmospheric pressure:	999,0 hPa
		Average deviation of measurements:	± 1.89 μm

Calibration certificate no. 09/2022

Leveling staff (type, no.):
GPLE3N,, no. 47068
Calibration range [m]:
0,12043 - 2,87934

Contract:
[redacted]
Date
3.9.2022

Corrections for the center of the right scale of the levelling staff [µm]:

	0	1	2	3	4	5	6	7	8	9
0										0.00
1	0.00	8.79	39.24	29.80	15.66	26.11	16.47	11.89	-4.78	-67.78
2	0.00	9.75	45.05	34.12	6.07	25.56	6.46	3.63	7.70	-65.71
3	0.00	14.78	51.37	44.91	8.95	35.11	17.83	16.75	-4.65	-72.72
4	0.00	17.34	47.51	39.95	18.45	29.51	13.42	22.34	0.41	-69.67
5	0.00	13.41	55.43	41.87	4.38	25.45	18.37	23.80	-1.15	-58.97
6	0.00	15.77	50.37	33.49	14.70	31.74	20.42	18.66	-1.19	-58.05
7	0.00	15.52	52.67	40.67	12.84	32.60	10.63	23.49	-8.75	-65.45
8	0.00	15.18	48.31	40.10	16.87	21.81	18.87	23.79	-1.27	-58.15
9	0.00	22.50	46.48	40.88	8.03	21.12	10.88	31.95	-8.58	-56.25
10	0.00	19.73	43.45	41.89	28.73	25.64	21.44	32.49	11.19	-50.98
11	2.06	19.47	40.39	24.48	27.78	22.07	15.76	26.68	-9.66	-52.36
12	1.32	18.35	27.61	40.18	20.45	15.95	21.48	21.37	-14.14	-60.55
13	-5.40	10.42	37.81	30.02	27.33	16.40	19.46	22.61	-22.89	-52.49
14	-7.52	19.49	36.36	25.35	27.03	19.28	5.13	24.35	-23.95	-55.96
15	-7.95	19.77	41.42	19.09	31.32	9.46	15.41	24.60	-36.08	-55.52
16	-6.78	26.45	42.72	19.96	24.39	4.43	6.30	27.31	-33.58	-49.74
17	-7.03	22.74	38.77	23.78	19.37	9.68	-3.42	26.12	-22.89	-50.72
18	-10.01	27.85	44.98	18.30	26.72	3.95	16.75	25.33	-34.99	-58.29
19	-11.28	25.00	46.42	17.73	13.37	-5.51	-4.72	27.08	-29.41	-62.05
20	-15.58	26.66	44.48	15.65	13.83	-8.73	5.89	24.27	-29.33	-53.68
21	-16.46	30.78	47.31	11.55	17.72	-3.01	17.57	35.73	-43.09	-61.79
22	-11.78	26.32	42.98	5.01	9.02	-5.04	3.22	34.14	-45.33	-51.36
23	-8.08	35.12	51.28	7.27	7.72	-0.51	-1.64	38.82	-52.82	-57.65
24	-3.25	36.28	52.68	5.83	25.96	3.09	4.67	37.79	-47.92	-48.10
25	2.80	39.63	47.68	6.15	23.93	4.15	11.76	41.64	-51.63	-37.89
26	1.68	40.48	49.99	10.00	32.21	-6.48	1.69	24.51	-49.25	-40.75
27	1.38	34.82	51.36	14.76	26.83	-2.79	10.43	29.99	-39.78	-48.91
28	-1.37	40.15	48.39	10.39	33.59	-7.53	11.56	26.85	-33.25	-38.27
29	-6.52	39.68	50.45	22.28	18.84	-12.46	11.43	21.30	-56.28	-33.92
30	-1.29	33.69	43.49	9.80	27.61	-5.82	3.51	30.33	-48.49	-32.02
31	-4.27	32.15	36.79	3.37	24.12	-19.43	10.46	36.07	-47.37	-30.76
32	-8.02	24.91	49.01	-2.36	19.53	-16.07	27.40	36.07	-53.81	-37.13
33	-8.89	33.99	43.87	10.26	25.98	-12.93	19.19	24.36	-55.44	-31.57
34	-18.53	30.06	31.97	13.72	29.61	-14.43	16.93	29.13	-57.90	-37.26
35	-17.67	30.94	33.71	-1.58	16.84	-24.69	9.71	27.96	-53.93	-35.66

Technical specialist: Ing. Pavol Kajánek, PhD. Bratislava, 3.9.2022

Laboratory director: doc. Ing. Ján Erdélyi, PhD. Institute director: prof. Ing. Alojz Kopáčik, PhD.

Calibration certificate no. 09/2022

Leveling staff (type, no.):
GPLE3N,, no. 47068
Calibration range [m]:
0,12043 - 2,87934

Contract:
[redacted]
Date
3.9.2022

Corrections for the center of the right scale of the levelling staff [µm]

	0	1	2	3	4	5	6	7	8	9
36	-15.35	27.92	40.51	2.97	18.68	-23.21	3.30	39.46	-52.54	-35.11
37	-19.59	25.77	38.87	11.19	25.82	-28.98	-5.33	41.14	-53.35	-34.05
38	-27.42	44.63	47.56	-6.44	26.68	-21.46	-2.42	31.31	-52.85	-44.03
39	-22.51	32.64	51.43	17.85	23.44	-20.02	-24.12	24.91	-57.84	-34.31
40	-23.19	24.60	45.15	3.59	26.45	-19.29	-2.24	9.67	-44.32	-47.93
41	-21.82	32.61	41.12	-5.32	29.18	-11.79	-1.25	22.16	-62.48	-33.80
42	-17.96	32.13	43.64	-9.53	26.83	-21.78	-20.54	21.53	-66.01	-20.84
43	-19.89	36.58	55.56	4.63	29.29	-7.01	-9.60	20.21	-73.07	-25.81
44	-16.00	43.97	52.26	4.57	33.10	-13.99	-8.51	22.23	-64.15	-8.86
45	-11.35	41.30	45.80	-4.84	25.99	-13.79	2.32	25.02	-57.92	-14.00
46	-6.50	43.66	48.24	3.57	31.58	-21.31	-16.26	31.50	-56.23	-11.28
47	-5.20	39.24	49.80	3.29	29.06	-8.68	3.79	23.85	-70.86	-19.60
48	-8.00	37.39	55.58	0.46	26.46	-3.29	9.89	29.41	-57.26	-13.49
49	-8.01	38.93	49.03	1.51	26.01	-0.44	7.80	31.34	-60.93	-17.54
50	-2.44	39.66	53.63	-2.85	24.75	-0.82	19.57	34.58	-54.74	-10.70
51	-0.44	37.58	52.07	3.68	27.91	-3.21	12.49	32.16	-56.74	-6.76
52	1.27	37.09	37.85	4.97	28.12	4.03	30.11	28.06	-64.40	-15.74
53	-2.75	33.69	40.26	8.01	31.75	10.23	18.89	25.69	-62.48	-13.14
54	-0.94	31.14	37.50	10.95	23.94	12.98	21.16	17.82	-64.16	-17.64
55	-5.51	31.57	31.28	14.55	28.58	20.64	13.70	16.79	-58.07	-13.30
56	1.11	36.13	51.44	21.29	24.01	3.65	2.08	15.38	-54.19	-16.39
57	-0.84	38.11	50.00	16.92	25.36	3.77	-6.52	15.87	-54.80	-12.03
58	5.55	41.09	43.35	18.37	30.37	9.80	15.43	12.73	-56.16	-16.45
59	9.77	34.67	46.56	13.52	31.72	-6.09	-15.12	4.33	-55.77	-10.67
60	11.41	37.35	41.76	14.70	37.63	8.16	6.37	-11.51	-75.88	-11.05

Technical specialist: Ing. Pavol Kajánek, PhD. Bratislava, 3.9.2022

Laboratory director: doc. Ing. Ján Erdélyi, PhD. Institute director: prof. Ing. Alojz Kopáčik, PhD.
