# Supplementary material for: Metrology of Short-Length Measurers—Development of a Comparator for the Calibration of Measurers Based on Image Processing and Interferometric Measurements
Source: Sensors (Basel). 2024 Feb 29;24(5):1573. doi: 10.3390/s24051573 (PMC10934804; doi:10.3390/s24051573)
Supplement: Supplementary file 1 [file sensors-24-01573-s001.zip › File S2-coded_scale.pdf]

Calibration certificate no. 01/2019

Leveling staff (type, no.):
GPCL3,, no. 32316
Calibration range [the order of the scale division]:
9-682
Comparator:
Horizontal comparator
Contract:
[redacted]
Date:
13.12.2018

Laboratory of metrology of short lengths
Slovak University of Technology in Bratislava
Faculty of Civil Engineering
Department of Surveying
Radlinského 11
810 05 Bratislava
tel. [redacted]
email: [redacted]

Coefficient of expansion (standardized value):
$\alpha_{T_N} = 0{,}50\ ppm \cdot °C^{-1}$

Position of the leveling staff during calibration:
horizontal

Determinaion of scale factor:

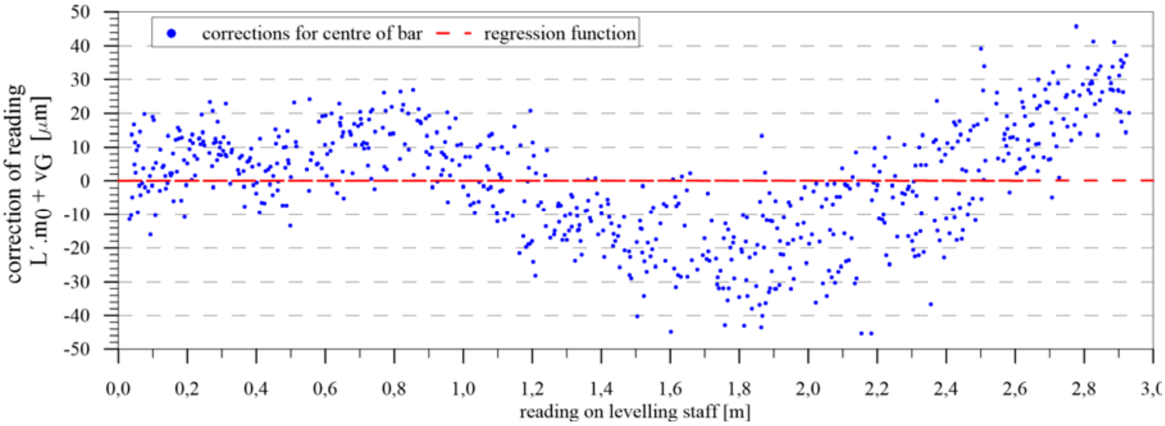

Scale factor: $\boldsymbol{m_0 = -0.04 \pm 0{,}02\ ppm}$ $\boldsymbol{T_0 = 20{,}2°C}$ $\boldsymbol{v_G = -34 \cdot 10^{-6}\ m}$

Reading on the levelling staff after correction:

$$L = L' \cdot [1 + m_0 + \alpha_T \cdot (T - T_0) \cdot 10^{-6}] + v_G$$

$L´[m]$	measured value	m_0	scale factor
$T[°C]$	temperature during measurement	$\alpha_T[\text{ppm} \cdot °C^{-1}]$	coeficient of expansion
$T_0[°C]$	temperature during calibration	$v_G[m]$	index correction

Determination of scale factor:

1st measurement:

scale factor:		Average temperature	20,4 °C
0.0341 ppm	± 0,020 ppm	Average atmospheric pressure:	1003.9 hPa
		Average deviation of measurements:	± 5.83 µm

2nd measurement:

scale factor:		Average temperature	20,0 °C
0.0417 ppm	± 0,036 ppm	Average atmospheric pressure:	1005,7 hPA
		Average deviation of measurements:	± 5,75 µm

Average from both measurement:

scale factor:		Average temperature	20,2 °C
0.0379 ppm.	± 0,028 ppm	Average atmospheric pressure:	1004.8 hPa
		Average deviation of measurements:	± 5.79 µm

Calibration certificate no. 01/2019

Leveling staff (type, no.):
GPCL3,, no. 32316

Calibration range [the order of the scale division]:
9-682

Contract:
[redacted]

Date
13.12.2018

Corrections for the centre of the division of the leveling staff scale [μm]:

	0	1	2	3	4	5	6	7	8	9
0										-11.44
10	-10.19	13.77	-4.88	16.66	4.83	11.38	2.32	9.17	-9.47	10.45
20	-1.41	14.61	-4.10	-0.72	3.64	19.77	-1.81	-3.10	1.35	-15.95
30	-1.35	18.92	3.71	17.93	-10.18	5.41	-2.84	9.24	3.99	4.59
40	1.48	-2.21	-0.66	13.31	2.16	1.51	2.19	8.12	-5.99	12.28
50	2.66	15.63	12.63	14.79	-5.85	12.86	6.11	-10.81	-1.19	5.19
60	9.37	12.13	0.30	13.72	11.28	7.54	1.60	6.44	12.63	12.74
70	10.87	19.55	14.39	11.68	10.59	8.26	23.36	4.31	20.72	-1.80
80	11.20	12.57	11.54	6.10	17.53	7.80	9.89	11.35	7.60	9.03
90	13.07	7.85	22.86	7.37	11.31	-0.85	8.54	9.26	3.16	2.12
100	6.86	7.45	2.85	-1.77	-7.62	5.61	6.06	6.76	9.53	4.17
110	-1.15	-3.73	-0.54	4.88	-9.35	12.52	-6.52	-6.59	2.94	3.40
120	-1.06	4.24	-4.24	1.33	15.16	11.13	5.24	-2.71	-0.18	13.26
130	-7.31	9.99	3.83	10.17	-7.23	14.69	6.23	5.14	-13.30	1.13
140	23.26	11.57	10.19	3.45	-2.90	24.14	6.14	8.35	8.07	4.91
150	5.12	17.44	3.78	19.09	10.70	19.25	1.37	7.93	9.00	20.01
160	-1.08	11.33	-3.56	22.84	17.09	16.31	18.58	13.24	2.70	14.37
170	-5.24	14.51	13.76	2.24	16.86	10.69	16.22	20.38	18.36	16.87
180	10.70	11.46	9.63	11.01	4.58	9.71	18.19	20.46	0.65	21.18
190	-2.43	14.51	15.66	26.16	4.12	20.73	3.34	13.96	21.91	13.99
200	6.43	26.46	20.95	22.56	10.87	20.02	9.29	27.07	1.76	17.77
210	21.22	17.51	17.05	18.45	8.58	13.35	3.93	19.24	7.87	9.89
220	-1.90	14.42	-0.20	1.79	8.42	3.27	7.86	11.65	6.94	20.30
230	-6.30	15.73	1.60	6.19	18.54	10.64	4.76	10.89	-1.75	7.08
240	-4.23	7.07	-7.12	-6.39	-9.55	10.18	-6.21	-0.43	10.75	-1.61
250	4.35	-2.35	3.35	13.36	-1.93	13.95	-7.39	9.67	-9.68	-12.01
260	1.92	-3.07	0.86	10.12	-2.72	-11.61	-9.66	8.31	-10.45	16.01
270	-21.64	10.64	-16.54	-5.99	-19.75	-0.19	-18.88	3.89	-18.79	20.69
280	-17.99	11.46	-24.21	7.54	-28.11	2.28	-7.33	-6.28	-8.55	1.50
290	-13.47	9.16	-17.00	-16.00	-14.84	-7.38	-6.60	-12.17	-7.28	-17.64
300	-16.11	-10.34	-17.67	-14.80	-9.97	-4.05	-13.73	-4.68	-23.95	-8.00
310	-10.40	-14.85	-14.48	-13.13	-17.88	-22.12	-8.85	-8.78	0.21	-5.87
320	-11.08	-11.99	0.79	-3.74	-16.02	-7.53	-9.82	-14.79	-22.77	-5.56
330	-6.78	-10.71	-12.76	-19.51	-15.83	-17.92	-16.49	-13.55	-25.67	-10.67
340	-20.83	-21.01	-27.97	-22.71	-28.99	-18.21	-40.22	-11.84	-14.13	-14.31
350	-21.32	-1.52	-34.23	-24.02	-26.96	-18.38	-22.54	-9.18	-20.46	-7.95

Technical specialist: Ing. Pavol Kajánek, PhD. Bratislava, 13.12.2018

Laboratory director: doc. Ing. Ján Erdélyi, PhD. Institute director: prof. Ing. Alojz Kopáčik, PhD.

Calibration certificate no. 01/2019

Leveling staff (type, no.):
GPCL3,, no. 32316

Contract:
[redacted]

Calibration range [the order of the scale division]:
9-682

Date
13.12.2018

Corrections for the centre of the division of the leveling staff scale [μm]:

	0	1	2	3	4	5	6	7	8	9
360	-25.51	-17.40	-12.75	-6.83	-23.92	-7.46	-44.85	0.37	-25.96	-4.70
370	-31.62	-7.52	-28.00	-6.61	-28.51	1.29	-13.42	-2.72	-28.46	2.15
380	-23.63	-26.29	-22.25	-17.15	-23.36	-3.63	-21.07	-23.86	-11.87	-23.21
390	-30.83	-28.55	-31.92	-26.60	-31.92	-17.08	-42.89	-12.64	-35.47	-14.44
400	-31.95	-19.88	-23.49	-18.41	-16.83	-29.00	-34.56	-20.35	-21.16	-10.31
410	-43.01	-18.19	-29.42	-10.95	-34.15	-22.88	-37.96	-21.74	-31.19	-19.08
420	-36.78	-6.12	-43.48	13.31	-40.16	-10.33	-19.91	2.36	-36.30	-11.79
430	-33.02	-3.58	-31.94	-11.89	-26.85	-12.13	-32.01	-5.91	-32.11	-14.11
440	-30.99	-18.01	-21.95	-22.11	-10.84	-18.78	-18.80	-6.36	-25.87	-7.22
450	-32.07	-17.66	-29.14	-13.09	-17.04	-5.13	-12.50	-1.90	-24.48	-18.80
460	-16.05	-3.01	-36.14	-0.93	-18.11	-7.58	-30.41	-5.39	-34.12	-1.12
470	-26.62	-14.65	-28.64	-6.90	-16.00	-0.91	-30.20	-4.71	-26.99	-4.62
480	-25.45	3.68	-15.66	-0.98	-24.02	-1.31	-23.66	-2.22	-17.18	1.13
490	-30.41	8.48	-28.92	-1.13	-45.34	-45.34	-5.65	-0.59	4.51	9.77
500	-2.63	-1.29	-11.18	2.71	-11.55	-10.06	-12.52	6.93	-22.24	12.83
510	-24.87	-1.37	-4.88	1.36	-16.66	-3.56	-6.60	10.72	-3.92	-2.33
520	-16.50	-1.71	-18.00	-3.08	-20.65	-0.27	-22.59	4.58	-15.90	-4.28
530	-22.30	9.87	-14.99	4.69	-9.80	13.51	-21.27	4.65	-36.73	11.54
540	-11.87	12.23	-5.10	23.75	-14.88	9.41	-17.85	5.90	-22.91	7.85
550	-11.19	11.20	-15.61	9.85	-8.42	17.39	-10.94	16.53	-17.63	11.70
560	-3.70	17.63	-7.96	13.63	-17.23	3.38	-14.61	2.95	-10.02	12.30
570	-6.83	14.79	-5.13	16.24	-11.75	16.61	3.08	39.20	-5.39	26.82
580	0.30	33.83	6.01	18.22	22.97	26.24	6.65	5.94	12.42	13.83
590	10.40	19.21	15.35	8.18	-3.39	12.84	12.99	16.69	2.29	12.47
600	6.48	27.21	3.41	23.41	3.67	21.70	4.02	19.20	9.21	12.09
610	16.32	16.67	7.19	25.24	10.90	21.41	2.94	29.94	11.03	22.16
620	7.02	27.22	23.44	23.15	6.39	28.89	6.45	17.16	-4.89	32.11
630	13.77	21.27	10.19	28.94	0.90	18.24	24.86	25.59	15.98	33.88
640	12.05	26.59	16.43	45.67	21.26	25.29	14.00	28.66	19.13	33.38
650	27.05	28.75	26.09	29.32	28.92	41.21	22.80	31.43	13.58	32.77
660	23.81	33.95	26.36	30.46	27.18	28.97	27.14	33.79	26.90	41.14
670	21.08	26.93	26.63	31.03	20.36	35.68	17.52	33.76	26.42	34.95
680	14.38	37.24	20.11							

Technical specialist: Ing. Pavol Kajánek, PhD. Bratislava, 13.12.2018

Laboratory director: doc. Ing. Ján Erdélyi, PhD. Institute director: prof. Ing. Alojz Kopáčik, PhD.
