# Supplementary material for: Metrology of Short-Length Measurers—Development of a Comparator for the Calibration of Measurers Based on Image Processing and Interferometric Measurements
Source: Sensors (Basel). 2024 Feb 29;24(5):1573. doi: 10.3390/s24051573 (PMC10934804; doi:10.3390/s24051573)
Supplement: Supplementary file 1 [file sensors-24-01573-s001.zip › File S3-measuring_tape.pdf]

Calibration certificate no. 03/2021

Calibrated measurer (type, no.):
30 m steel measuring band on metal - plastic fork,
no. 3-S12-3005 U-1
Calibration range [m]:
0.1 - 29.1
Comparator:
Horizontal comparator
Contract:
[redacted]
Date:
8.3.2021

Laboratory of metrology of short lengths

Slovak University of Technology in Bratislava
Faculty of Civil Engineering
Department of Surveying
Radlinského 11
810 05 Bratislava
[redacted]
email [redacted]

coefficient of expansion (standardized value for steel)
$\alpha_{T_N} = 11\,ppm \cdot °C^{-1}$

Position of the measuring tape during calibration: horizontal

Determinaion of scale factor:

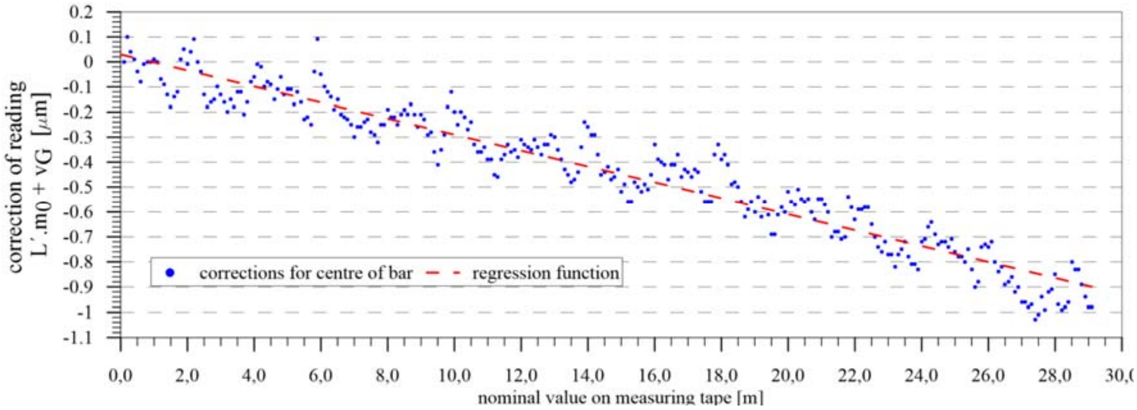

Scale factor: $\boldsymbol{m_0 = -0,03 \pm 0,0014\,ppm}$ $\boldsymbol{T_0 = 19,8°C}$

Reading on the levelling staff after correction:

$$L = L' \cdot [1 + m_0 + \alpha_T \cdot (T - T_0) \cdot 10^{-6}]$$

$L'[m]$	measured value	m_0	scale factor
$T[°C]$	temperature during measurement	$\alpha_T[\text{ppm} \cdot °C^{-1}]$	coefficient of thermal expansion of steel
$T_0[°C]$	temperature during calibration		

Determination of scale factor:
1st measurement:
scale factor:
-0.0250 ppm ± 0,001 ppm

Average temperature 20,2 °C
Average atmospheric pressure: 1016.4 hPa
Average deviation of measurement: ± 0.05 mm

2nd measurement:
scale factor:
-0.0388 ppm ± 0,001 ppm

Average temperature 19,4 °C
Average atmospheric pressure: 1016.0 hPa
Average deviation of measurement: ± 0.05 mm

Average from both measurement:
scale factor:
-0.032 ppm. ± 0,001 ppm

Average temperature 19,8 °C
Average atmospheric pressure: 1016.2 hPa
Average deviation of measurement: ± 0.05 mm

Calibration certificate no. 03/2021

Leveling staff (type, no.):
30 m steel measuring band on metal - plastic fork,
no. 3-S12-3005 U-1

Contract:
[redacted]

Calibration range [m]:
0.1-29.1

Date
8.3.2021

Corrections for the centre of the bar from the measuring tape [mm]:

m\dm	0	1	2	3	4	5	6	7	8	9
0	-	0.00	0.10	0.04	0.01	-0.04	-0.08	-0.01	0.00	0.00
1	0.01	0.00	-0.07	-0.09	-0.13	-0.18	-0.14	-0.12	0.01	0.05
2	-0.01	0.04	0.09	0.00	-0.04	-0.13	-0.18	-0.16	-0.15	-0.10
3	-0.13	-0.16	-0.20	-0.15	-0.18	-0.12	-0.12	-0.21	-0.16	-0.08
4	-0.06	-0.01	-0.02	-0.10	-0.08	-0.09	-0.15	-0.11	-0.06	-0.13
5	-0.11	-0.11	-0.17	-0.12	-0.16	-0.23	-0.22	-0.25	-0.04	0.09
6	-0.05	-0.10	-0.12	-0.14	-0.19	-0.15	-0.21	-0.22	-0.23	-0.25
7	-0.30	-0.26	-0.26	-0.24	-0.23	-0.28	-0.29	-0.32	-0.25	-0.25
8	-0.19	-0.22	-0.22	-0.25	-0.21	-0.19	-0.21	-0.17	-0.21	-0.26
9	-0.21	-0.23	-0.29	-0.28	-0.36	-0.41	-0.35	-0.29	-0.18	-0.12
10	-0.20	-0.25	-0.20	-0.22	-0.27	-0.24	-0.33	-0.36	-0.36	-0.34
11	-0.39	-0.39	-0.45	-0.46	-0.39	-0.37	-0.33	-0.36	-0.35	-0.38
12	-0.31	-0.33	-0.34	-0.35	-0.31	-0.34	-0.37	-0.33	-0.33	-0.29
13	-0.30	-0.35	-0.39	-0.43	-0.45	-0.48	-0.47	-0.44	-0.34	-0.24
14	-0.26	-0.29	-0.29	-0.37	-0.45	-0.44	-0.42	-0.47	-0.46	-0.43
15	-0.52	-0.49	-0.56	-0.56	-0.48	-0.50	-0.52	-0.49	-0.51	-0.45
16	-0.33	-0.39	-0.40	-0.41	-0.47	-0.41	-0.41	-0.37	-0.46	-0.43
17	-0.44	-0.46	-0.43	-0.44	-0.52	-0.56	-0.56	-0.56	-0.37	-0.33
18	-0.39	-0.37	-0.41	-0.49	-0.48	-0.50	-0.56	-0.62	-0.59	-0.56
19	-0.60	-0.54	-0.62	-0.56	-0.61	-0.69	-0.69	-0.61	-0.58	-0.60
20	-0.52	-0.56	-0.57	-0.51	-0.55	-0.56	-0.55	-0.60	-0.63	-0.55
21	-0.55	-0.57	-0.60	-0.70	-0.68	-0.68	-0.71	-0.70	-0.54	-0.58
22	-0.63	-0.59	-0.59	-0.58	-0.58	-0.65	-0.70	-0.74	-0.76	-0.72
23	-0.77	-0.77	-0.82	-0.77	-0.75	-0.72	-0.78	-0.81	-0.81	-0.83
24	-0.72	-0.71	-0.66	-0.64	-0.69	-0.73	-0.72	-0.72	-0.74	-0.71
25	-0.76	-0.78	-0.78	-0.80	-0.75	-0.83	-0.90	-0.88	-0.74	-0.73
26	-0.74	-0.72	-0.80	-0.84	-0.82	-0.89	-0.88	-0.86	-0.92	-0.90
27	-0.96	-0.96	-0.98	-0.97	-1.03	-1.01	-0.94	-0.99	-0.92	-0.91
28	-0.85	-0.97	-0.99	-0.98	-0.96	-0.80	-0.83	-0.83	-0.89	-0.94
29	-0.98	-0.98	-	-	-	-	-	-	-	-

note: correction of the reading on the measuring tape can be realized in two ways, namely (1) using the relationship from the first page of this protocol or (2) adding a correction directly interpolated from the correction table mentioned above.

Technical specialist: Ing. Pavol Kajánek, PhD. Bratislava, 8.3.2021

Laboratory director: doc. Ing. Ján Erdélyi, PhD. Institute director: prof. Ing. Alojz Kopáčik, PhD.
